# Geologically-inspired and mutation-aware driven well log density reconstruction method for complex heterogeneous formations

**DOI:** 10.1371/journal.pone.0347378

**Published:** 2026-10-13

**Authors:** Yang Li, Haiwei Guo, Xianfeng Gong, Ping Liu, Ning Hao, Yuping Meng

**Affiliations:** 1 Zhongyuan Oilfield Informatization Management Center Department, Sinopec, Puyang, China; 2 Zhongyuan Oilfield Company, Sinopec, Puyang, China; Northeastern University, CHINA

## Abstract

To address the issue of data distortion in traditional well log density reconstruction methods caused by harsh borehole environments in complex heterogeneous formations, and the challenge for general machine learning models to capture high-frequency abrupt change features due to the lack of geophysical constraints, this paper proposes a density curve reconstruction framework integrating geologically-inspired feature engineering and a mutation-aware mechanism. The method first constructs a multi-dimensional enhanced feature space covering sedimentary cycle characteristics and fluid properties through multi-scale geologically-inspired analysis, utilizing robust range normalization to eliminate interference from outliers. Subsequently, a mutation-aware driven algorithm based on the gradient change rate is innovatively designed. By dynamically adjusting loss weights, it resolves the drawback where traditional smoothing algorithms tend to erase formation interface information. Finally, geological prior constraint terms are embedded into the XGBoost architecture to achieve multi-objective optimization that balances data-driven accuracy with consistency in geological laws. Under leave-one-well-out cross-validation over ten wells, the proposed method achieves a mean R² of 0.98, an RMSE of 0.029, and an MAE of 0.022. Experimental results demonstrate that the proposed method not only improves reconstruction accuracy but also significantly enhances the physical consistency of the results. Furthermore, it precisely identifies thin interbedded boundaries, providing highly reliable basic data support for the refined evaluation of oil and gas reservoirs.

## 1. Introduction

Well log data serve as a pivotal bridge connecting seismic information with geological interpretation, playing an essential role in reservoir evaluation and fluid identification [1–3]. However, key logging curves—particularly density (DEN)—frequently suffer from data gaps or unreliable quality caused by adverse borehole conditions, instrument malfunctions, and operational costs [4], a problem especially acute in thin interbeds, fractured zones, and other complex heterogeneous formations, where logging responses become strongly non-stationary with high-frequency step-like characteristics [5]. In such settings, traditional global normalization is highly sensitive to washout-induced outliers, while general machine-learning models that take only raw curves as input tend toward “black-box” mapping: lacking implicit physical constraints, they generalize poorly in unknown intervals and may yield reconstructions that violate petrophysical laws [6].

Existing methods for reconstructing missing well logs can be broadly grouped into four families: empirical-formula and statistical models (Gardner, RLOESS, MLR/SVM/GPR) [7–11]; recurrent-network methods (LSTM/GRU and their bidirectional variants, Bi-LSTM/Bi-GRU) [12–17]; attention and Transformer architectures [18]; and ensemble-learning and generative hybrids (Random Forest, XGBoost, GAN, VAE) [19–23]. While these approaches have achieved progress in their respective settings, most do not explicitly embed petrophysical mechanisms and geological spatial-evolution laws as core constraints within the model framework, and therefore remain prone to over-smoothing and loss of high-frequency step signals at strongly heterogeneous interfaces such as thin interbeds and fractured zones.

To address the aforementioned issues, a geologically-inspired and mutation-aware driven well log density reconstruction method for complex heterogeneous formations is proposed. It should be emphasized that the novelty lies not in any individual module — gradient boosting, petrophysical feature construction, robust normalization, and gradient-based boundary detection are all established — but in a single shared mutation-aware mechanism that runs end-to-end through the model, preserving the geological step signals that uniform filters and conventional regressors tend to erase. The specific contributions are as follows:

(1) A theoretically grounded mutation operator as the backbone. A multi-curve gradient-fusion score (the comprehensive mutation score, Eq. 11), computed from the reference set (GR, AC, RT), serves as a one-dimensional discrete geological structural-tensor descriptor; by fusing gradients across curves it separates genuine lithological boundaries from washout-induced single-curve spikes without any external lithology label.
(2) A shared gradient/mutation-aware mechanism running through both input and output (perceive-then-preserve). The same gradient-based mutation-sensing principle is embedded on both sides of the model: on the input side as per-curve spatial-evolution features (first-order differences and local anomaly scores, Eqs. 7–9) that let the regressor perceive interfaces, and on the output side as their multi-curve fusion $W_{smooth}\left(t\right)$ (Eqs. 11–13), which preserves interfaces via zero-smoothing where mutation is strong. This input–output coupling, rather than a loose concatenation, is the methodological core.
(3) An interface-aware optimized objective with a standard XGBoost learner. Without altering XGBoost’s tree learner, $W_{smooth}\left(t\right)$ is written into Eq. 14 as the weight of an inter-depth spatial-smoothness penalty added to the data-fidelity term, and the objective is optimized through a custom objective function; the optimization is thereby geology-aware, and the reconstructed density suppresses washout noise while retaining stratigraphic step signals, improving accuracy and physical consistency together.

The remainder of this paper is organized as follows: Section 2 details the methodology of integrating geological inspiration and mutation awareness from well logs into the machine learning model; Section 3 introduces the datasets, applications, and methodological comparisons, followed by a discussion and analysis of the results; and finally, Section 4 summarizes the research conclusions.

## 2. Methods

This section first defines the research objects focusing on complex heterogeneous reservoirs in the Zhongyuan Oilfield and outlines the overall technical route that integrates geological priors with data-driven approaches. Subsequently, from the three dimensions of feature system construction, mutation-aware logic, and model optimization architecture, a deep analysis is provided on how petrophysical mechanisms are transformed into computable constraint operators to achieve high-precision and physically consistent density reconstruction.

The research subjects of this study are well logging datasets from complex heterogeneous formations (e.g., thin interbeds and fractured zones) in the Zhongyuan Oilfield. Addressing the unreliable quality of density (DEN) curves caused by factors such as borehole enlargement and instrument failure in practical operations, this research utilizes fundamental logging responses—including depth (DEPTH), gamma ray (GR), acoustic transit time (AC), resistivity (RT), caliper (CAL), and compensated neutron (CNL)—to establish a physically consistent deep learning reconstruction scheme. The overall technical route is summarized in Fig 1, as detailed below:

**Fig 1:**
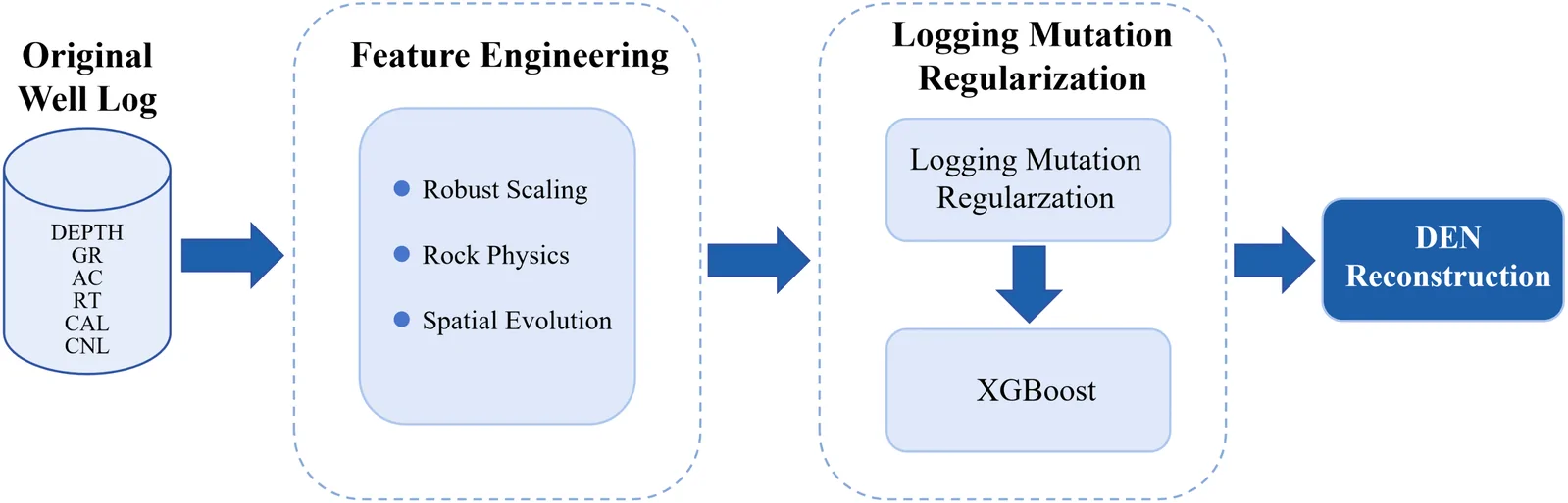
Overall technical flowchart.

(1) Preprocessing and construction of geologically-inspired feature engineering: Robust range normalization is employed to eliminate environmental noise, and petrophysical features such as shale content and porosity, along with spatial evolution features reflecting sedimentary cycles, are explicitly extracted. This process transforms static logging responses into multi-dimensional dynamic feature vectors containing geological priors, significantly enhancing the model’s perception of heterogeneous scenarios.
(2) Multi-dimensional gradient-driven mutation-aware smoothing mechanism: Smoothing weights are dynamically calculated by fusing multi-dimensional log gradient information to establish a logging mutation-aware regularization. This mechanism follows the logic of “strong mutation, weak smoothing,” effectively suppressing noise in homogeneous intervals while implementing “zero-smoothing” at lithological interfaces to precisely preserve critical geological step signals.
(3) Geologically-constrained XGBoost density reconstruction architecture: Multi-dimensional geological features are integrated as model inputs, and a spatial regularization term based on mutation-aware weights is introduced into the loss function to construct a multi-objective optimization function. Through the deep integration of data-driven frameworks and geological structures, physical consistency in density reconstruction is achieved within complex wellbore environments.

### 2.1. Data preprocessing and geologically-inspired feature engineering

To enhance the generalization capability of machine learning models in complex heterogeneous formations, three feature engineering systems were constructed in this study: well log data normalization, petrophysical features, and spatial evolution features. The technical route is illustrated in Fig 2.

**Fig 2:**
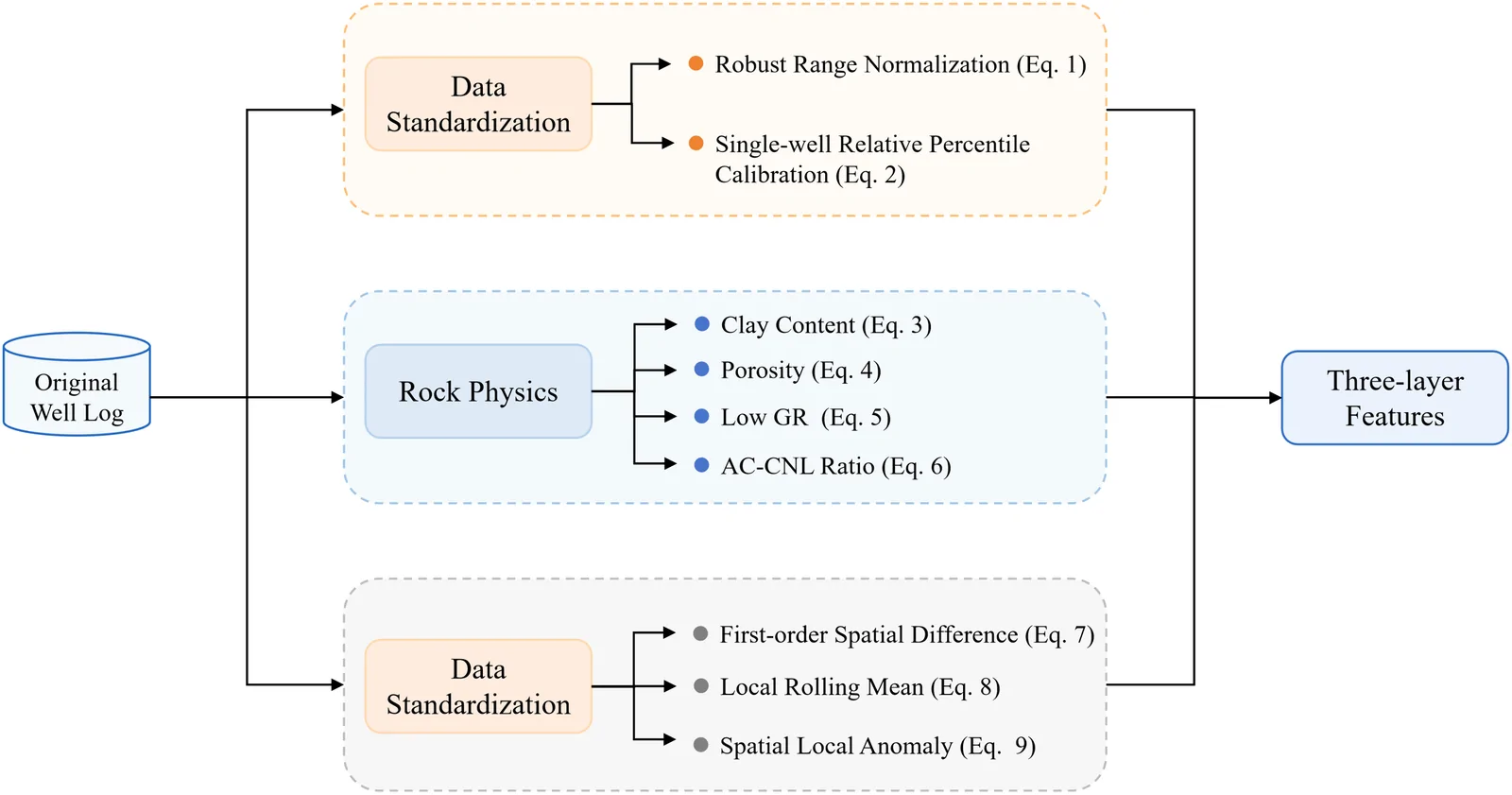
Technical flowchart of data preprocessing and geologically-inspired feature engineering.

#### 2.1.1. Well log data normalization.

In practical logging operations, data are frequently affected by borehole enlargement, mud filtrate invasion, differences in instrument models, and calibration biases, leading to baseline drift between different wells. Traditional global 0–1 normalization is extremely sensitive to extreme outliers (such as spikes caused by washouts), which tends to obscure authentic formation characteristics.

This study introduces a robust range normalization method. By extracting the 5th and 95th percentiles of single-well data for truncation and leveling, measurement noise caused by non-geological factors is effectively eliminated. The formula is as follows:


$$\begin{array}{c} X_{norm}=min\left(max\left(\frac{X-P_{05}\left(X\right)}{P_{95}\left(X\right)-P_{05}\left(X\right)},0\right),1\right) \end{array}$$ (1)

where X represents the original logging response value, denoting the physical parameter sequence to be processed; P_05_(X) is the 5th percentile of the variable in the single-well sequence, serving as the lower truncation point to filter low-value random noise; and P_95_(X) is the 95th percentile, acting as the upper truncation point to eliminate extreme abnormal spikes caused by environmental factors such as borehole enlargement. X_norm_ is the normalized feature value, whose distribution is mapped into a more robust scale space.

Furthermore, relative percentile ranking is introduced to reflect the relative development degree of rock pores within specific well intervals, thereby eliminating the absolute influence of formation depth on amplitude:


$$\begin{array}{c} X_{rank}=\frac{Rank\left(X_{i}\right)}{N} \end{array}$$ (2)

where Rank(X_i_) denotes the rank of the measured value at point i after sorting the entire well sequence in ascending order, and N is the total number of effective measured points in the well. The relative percentile feature X_rank_ reflects the relative development intensity (e.g., porosity quality) of the formation across the entire well, effectively eliminating amplitude bias introduced by absolute depth.

#### 2.1.2. Petrophysical mechanism-guided feature engineering.

To address this, by pre-constructing shale content and porosity features, the complex coupled responses are decomposed into independent physical dimensions, thereby reducing the search space for nonlinear models such as XGBoost.

The shale content feature (V_sh_) is utilized to constrain the matrix components of the formation and eliminate the interference of shale on the density response [24,25]. The formula is as follows:


$$\begin{array}{c} V_{sh}=\frac{GR-GR_{min}}{GR_{max}-GR_{min}} \end{array}$$ (3)

where GR is the measured value at depth t. GR_min_ is the baseline value for pure sandstone (or low-radioactivity matrix) within the study area, and GR_max_ is the baseline value for pure mudstone. V_sh_ is the dimensionless estimated shale content used to characterize lithological purity.

Petrophysical porosity features are derived from measurements across different physical dimensions to infer pore development, providing critical space-occupancy constraints for density reconstruction. This study employs the sonic porosity feature based on the Wyllie time-average equation [26,27]:


$$\begin{array}{c} POR_{AC}=\frac{AC-\Delta t_{ma}}{\Delta t_{f}-\Delta t_{ma}} \end{array}$$ (4)

where ∆t_ma_ represents the sonic interval transit time of the sandstone matrix, typically 180\hspace{0.33em}μs/m for clean sandstone. ∆t_f_ represents the sonic interval transit time of the fluid, where 620\hspace{0.33em}μs/m corresponds to the acoustic velocity in water.

The GR curve is a key basis for lithology identification. Governed by the enrichment of radioactive elements in clay minerals, high GR values typically reflect mudstone or high-shale formations, while low GR values correspond to “clean” formations with well-developed porosity, such as pure sandstone or carbonates [28,29]. To intuitively characterize reservoir quality, a low-GR indicator was constructed. This index quantifies formation “purity” through logical inversion: a value closer to 1 indicates higher lithological purity and superior reservoir quality, while a value closer to 0 indicates increased shale content. The calculation formula is expressed as:


$$\begin{array}{c} GR_{low\_proxy}=1-GR \end{array}$$ (5)

The sonic-neutron ratio feature significantly amplifies identification signals for gas-bearing layers or specific lithofacies, enhancing the model’s sensitivity to formation mutations [30]. Therefore, this study establishes the sonic-neutron ratio (R_AC_CNL_) to identify heterogeneous features that are difficult to capture with a single curve. The formula is as follows:


$$\begin{array}{c} R_{AC\_CNL}=\frac{AC}{CNL} \end{array}$$ (6)

#### 2.1.3. Spatial evolution features.

Formation sedimentation is a cyclic process (e.g., retrogradational and progradational sequences) influenced by tectonic movements and global sea-level changes, which determines that well logs possess strong spatial autocorrelation along depth. Traditional machine learning models typically treat each sampling point as an independent event, resulting in the loss of critical geological trend information. To capture the local variations of these sedimentary cycles, this study introduces temporal differencing and local anomaly statistics based on a predefined depth window W. This approach aims to transform single-point static features into dynamic features that reflect formation evolution trends.

First-order spatial difference features are primarily utilized to capture formation interfaces and lithological mutation points. The formula is as follows:


$$\begin{array}{c} \Delta X\left(t\right)=X\left(t\right)-X(t-1) \end{array}$$ (7)

where t is the sampling index representing the current vertical depth position; X(t) is the normalized logging response at depth t; X(t-1) is the response value at the previous sampling point (typically 0.125 m above). ∆X(t) is the instantaneous gradient feature, reflecting the rate of change in logging signals with depth, which is crucial for identifying thin interbeds and abrupt surfaces.

Local rolling mean is employed to extract the background field evolution trend of formations and smooth high-frequency random interference. The formula is as follows:


$$\begin{array}{c} \mu_{rolling}\left(t\right)=\frac{\text{1}}{W}\sum_{i=t-W/2}^{t+W/2}X\left(i\right) \end{array}$$ (8)

where W is the depth statistical window size. Its value is typically determined based on the bedding development in the study area. In this paper, the window size W was set to 1.5 m, corresponding to the minimum identifiable thickness of sedimentary cycles in the study area. i is the local depth index within the window. $\mu_{rolling}\left(t\right)$ is the average response level within the local depth interval, representing the baseline material composition of the sedimentary environment in that section.

Local spatial anomaly score features are used to quantify the intensity of formation heterogeneity, serving as a key indicator for identifying “abnormal signals” in complex reservoirs:


$$\begin{array}{c} S_{anomaly}\left(t\right)=\frac{\left|X\left(t)-\mu_{rolling}(t\right)\right|}{\sigma_{rolling}\left(t\right)} \end{array}$$ (9)

where $\sigma_{rolling}\left(t\right)$ is the standard deviation of the logging response within the depth window W, reflecting the original degree of heterogeneity in the formation interval. $\left|X\left(t)-\mu_{rolling}(t\right)\right|$ is the absolute deviation, measuring the extent to which the current measurement deviates from the background trend. S_anomaly_(t) is the normalized anomaly score. In high-heterogeneity formations (such as fractured zones or conglomerate intervals), this value increases significantly, assisting XGBoost in capturing geological details that might otherwise be obscured by the mean.

### 2.2. Mutation-aware drive for well log curves

In heterogeneous formations (e.g., thin sand beds, fractured zones, or abrupt lithological interfaces), well log curves exhibit strong non-stationarity and high-frequency step-like characteristics. Traditional machine learning reconstruction methods or global smoothing algorithms typically employ a uniform kernel function, which often leads to “over-smoothing.” This phenomenon inadvertently erases geologically significant step signals while eliminating numerical noise, resulting in blurred reservoir boundaries and subsequently affecting reserve evaluation. To address this, a “mutation-aware” mechanism was designed in this study. By calculating the gradient field of reference baseline curves, the smoothing weight is dynamically adjusted. This logic is equivalent to imposing an adaptive spatial regularization constraint based on a geological structural tensor on the regression model. This is more than an analogy. Because a lithological interface is physically a *simultaneous* contrast in mineralogy, porosity, and fluid, a genuine boundary induces coherent, co-depth jumps across multiple logs (GR, AC, RT), whereas non-geological artifacts such as borehole washout or tool spikes typically perturb only a single curve. Fusing the gradients of multiple normalized logs therefore constructs a one-dimensional discrete geological structural tensor that measures the strength of coherent structural change at each depth, and modulating the regularization strength inversely by this tensor is the depth-domain analog of structure-tensor-driven anisotropic, edge-preserving diffusion: regularization is suppressed where structure is strong (boundaries preserved) and permitted where structure is weak (noise removed). The mutation-aware mechanism is thus a theoretically grounded, boundary-preserving anisotropic regularization rather than an empirical filter. The technical flowchart of this regularization constraint is illustrated in Fig 3.

**Fig 3:**
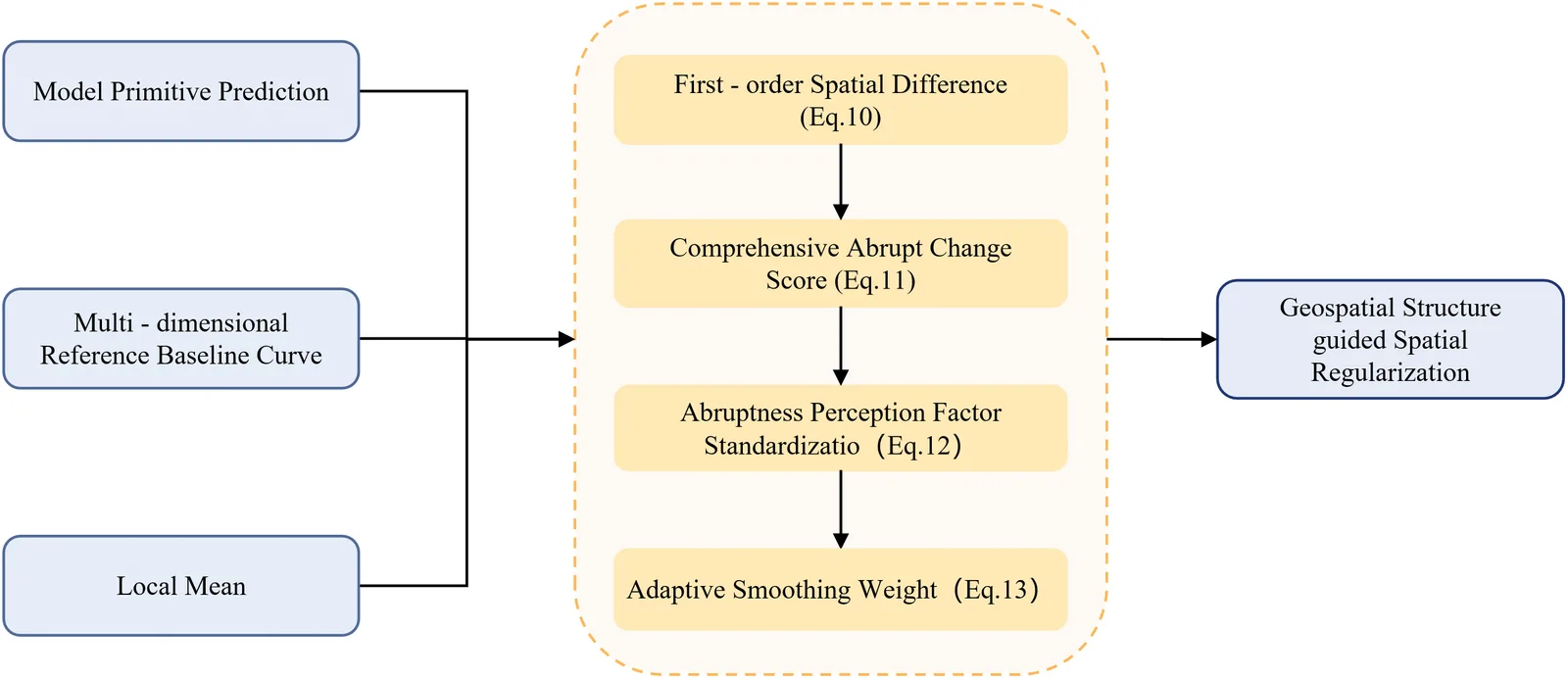
Technical flowchart of mutation-aware regularization for well log curves.

To accurately capture formation interfaces, a set of reference baseline curves [14] was defined to facilitate multi-dimensional gradient fusion and feature enhancement. For each depth sampling point t, the spatial gradient variation of each feature dimension was calculated. Specifically, the first-order absolute gradient (Eq. 10) and the comprehensive mutation score (Eq. 11) were employed:


$$\begin{array}{c} \nabla X_{k}\left(t\right)=\left|X_{k}\left(t)-X_{k}(t-1\right)\right| \end{array}$$ (10)

where k represents the reference feature index (k in C); X_k_(t) denotes the response value of the k-th reference feature (e.g., normalized GR) at depth t; and **∇**X_k_(t) reflects the instantaneous vertical variation intensity of that feature.


$$\begin{array}{c} S_{anomaly}\left(t\right)={max}_{k\in C}\left\{\nabla X_{k}(t)\right\} \end{array}$$ (11)

where S_anomaly_(t) represents the initial anomaly score. Taking the maximum gradient across multi-dimensional features ensures that the model perceives a potential geological mutation interface as long as a drastic change occurs in at least one physical parameter.

To eliminate the influence of different physical dimensions, the scores were mapped to the range [0, 1] as follows:


$$\begin{array}{c} {\overset{―}{S}}_{anomaly}\left(t\right)=\frac{S_{anomaly}\left(t\right)-min\left(S\right)}{max\left(S\right)-min\left(S\right)} \end{array}$$ (12)

where ${\overset{―}{S}}_{anomaly}\left(t\right)$ is the final mutation-aware intensity. A value closer to 1 indicates stronger formation heterogeneity or a distinct lithological mutation surface, while a value closer to 0 represents a relatively homogeneous formation.

Based on the normalized score, a nonlinear mutation-aware weight allocation function W_smooth_(t) was designed, following the principle of “strong mutation, weak smoothing.” The objective is to achieve adaptive smoothing weights using the following formula:


$$\begin{array}{c} W_{smooth}\left(t\right)=\left\{ \begin{array}{c} @l@0,\text{\textbackslash{}hspace\{1em}\;\;\;\;\;\;\}if\text{\textbackslash{}hspace\{0.33em}\}{\overset{―}{S}}_{anomaly}\left(t\right)\geq \lambda \\ 1-{\overset{―}{S}}_{anomaly}\left(t\right)\;,\text{\textbackslash{}hspace\{0.33em}\}if\;{\overset{―}{S}}_{anomaly}\left(t\right)<\lambda \end{array}\right. \end{array}$$ (13)

where λ is the mutation identification threshold. When the mutation intensity exceeds this threshold, the system identifies a definite geological interface. $W_{smooth}\left(t\right)$ serves as the spatial smoothing weight; when a strong formation mutation occurs, the weight drops toward 0, meaning the model fully preserves the original predicted values without smoothing.

Rather than acting as a post-hoc filter, this mutation-aware weight is embedded as the coefficient of a spatial-smoothness penalty in the optimized objective (Section 2.3), so that boundary preservation is achieved during optimization.

### 2.3. Geologically-constrained XGBoost density reconstruction architecture

In complexing heterogeneous formations, the relationship between well log curves and formation density is not a simple linear one; rather, it is a high-dimensional nonlinear mapping governed by the combined effects of lithology, pore structure, and fluid properties [31]. Well log responses in complex formations exhibit distinct stage-wise and mutational characteristics. Through the concatenation of multiple Classification and Regression Trees (CART), XGBoost [32] can accurately capture step-like features at formation interfaces, making it suitable for non-stationary well log curves. In practical production, multi-collinearity exists among logging features (e.g., the correlation between AC and CNL). The column-sampling mechanism of XGBoost effectively prevents model overfitting to a single sensitive curve, thereby enhancing the robustness of reconstructed curves in intervals with borehole enlargement. The technical route is illustrated in Fig 4.

**Fig 4:**
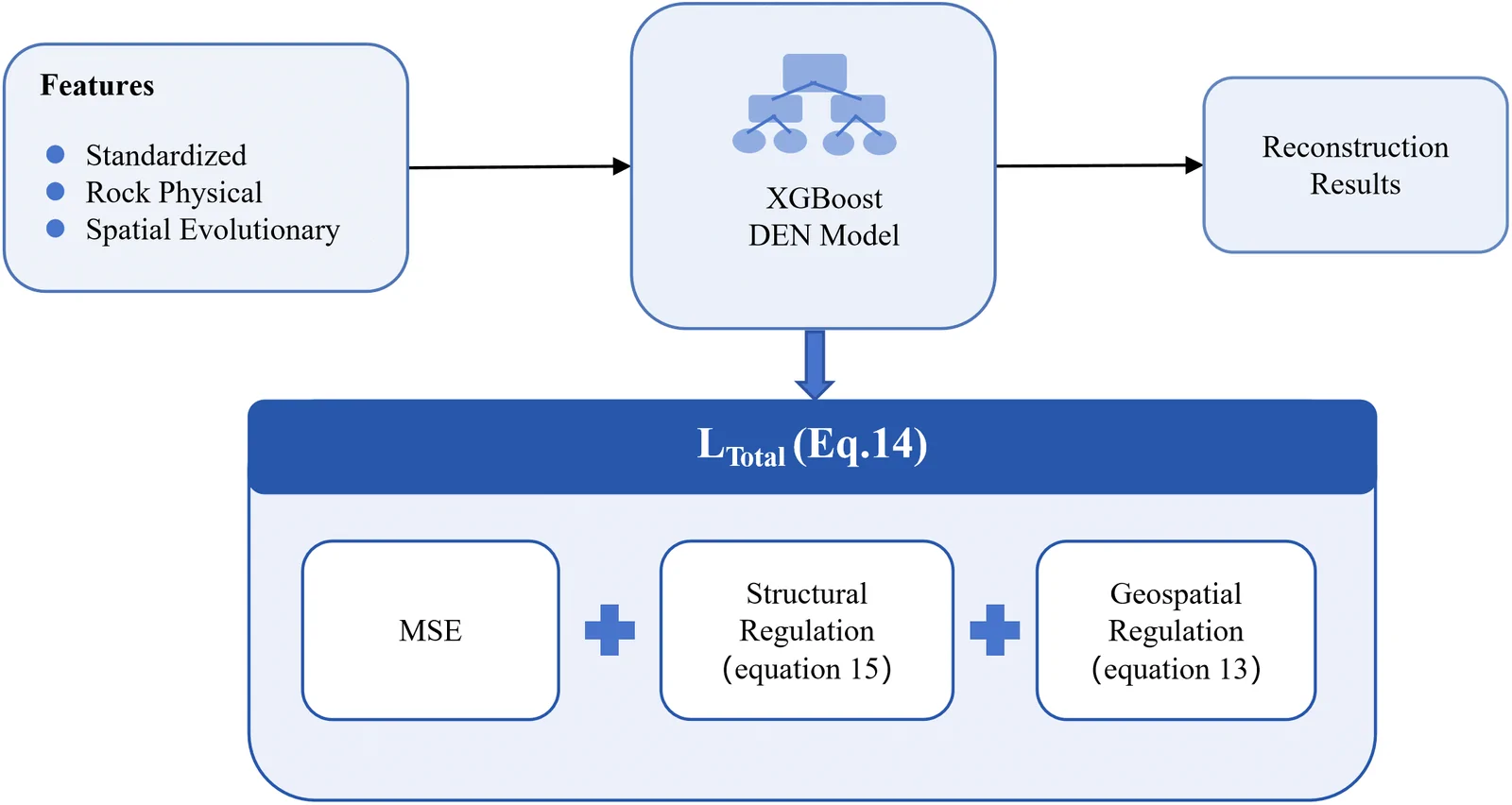
Technical flowchart of the geologically-constrained XGBoost density reconstruction architecture.

Diverging from general regression tasks, the input vector of the model not only includes raw physical quantities but also deeply integrates the geological information constructed in Section 2.1, ensuring the model possesses geological prior knowledge with a total of 30 features. These primarily include: fundamental response features (X_norm_, X_rank_) providing basic physical feedback; petrophysical feature variables (V_sh_, GR_low_proxy_, POR_AC_, R_AC_CNL_) explicitly injecting fluid property information; and spatial evolution, local smoothing, and abnormal mutation features (**∆**X, $\mu_{rolling}$, S_anomaly_), which empower the model to perceive sedimentary cycles and local heterogeneity.

To achieve physically consistent reconstruction, the total objective is an additive multi-objective loss combining data fidelity, the mutation-aware spatial-smoothness penalty, and the structural regularization:


$$\begin{array}{c} L_{\text{total}}=L_{\text{data}}+\lambda_{s}\text{\textbackslash{}hspace\{0.17em}\}L_{\text{smooth}}+\Omega \left(f\right) \end{array}$$ (14)

The data-fidelity term is the squared error over all N training depths:


$$\begin{array}{c} L_{\text{data}}=\sum_{i=1}^{N}{\left({\hat{y}}_{i}-y_{i}\right)}^{\text{2}} \end{array}$$ (15)

Where ${\hat{y}}_{i}$ is the reconstructed density and $y_{i}$ the measured label at depth i. The mutation-aware spatial-smoothness penalty reuses $W_{\text{smooth}}$ from Eq. 13 as the weight on inter-depth jumps:


$$\begin{array}{c} L_{\text{smooth}}=\sum_{i=1}^{N}\delta_{i}\text{\textbackslash{}hspace\{0.17em}\}W_{\text{smooth}}\left(t_{i}\right){\left({\hat{y}}_{i}-{\hat{y}}_{i-1}\right)}^{\text{2}},\text{\textbackslash{}hspace\{1em}\;\}\delta_{i}=\left\{ \begin{array}{cc} @ll@1, & \text{depths}i,\text{\textbackslash{}hspace\{0.17em}\}i-1\text{in the same well}\\ 0, & \text{otherwise} \end{array}\right. \end{array}$$ (16)

where $\lambda_{s}$ balances data fidelity against spatial consistency, and the indicator $\delta_{i}$ confines the difference operator to within a single well and breaks it at well boundaries. Through Eq. 16, the weight defined in Eq. 13 directly governs the optimization: at interfaces ($W_{\text{smooth}}\to 0$) the penalty vanishes and steps are preserved, whereas in homogeneous zones ($W_{\text{smooth}}\to 1$) inter-depth jumps are penalized and noise is suppressed. The reconstructed value is the additive output of K regression trees:


$$\begin{array}{c} {\hat{y}}_{i}=\sum_{k=1}^{K}f_{k}\left({𝐱}_{i}\right) \end{array}$$ (17)

where f_k_(x_i_) is the sum of predicted values from K decision trees, representing the final reconstruction result of the formation density by the model.


$$\begin{array}{c} \Omega \left(f\right)=\gamma T+\frac{\text{1}}{\text{2}}\lambda_{reg}\sum_{j=1}^{T}w_{j}^{\text{2}} \end{array}$$ (18)

with T the number of leaf nodes, $w_{j}$ the leaf scores, and $\gamma ,\lambda_{reg}$ penalty coefficients. Because $L_{\text{smooth}}$ couples adjacent depths, Eq. 14 cannot be optimized by per-sample weighting; instead it is realized through a custom objective that supplies, at each boosting round, the gradient $g_{i}$ (first-order derivative) and Hessian $h_{i}$ (second-order derivative) of $L_{\text{data}}+\lambda_{s}L_{\text{smooth}}$ with respect to the current prediction ${\hat{y}}_{i}$:


$$\begin{array}{c} g_{i}=\frac{\partial \left(L_{\text{data}}+\lambda_{s}L_{\text{smooth}}\right)}{\partial {\hat{y}}_{i}}=2\left({\hat{y}}_{i}-y_{i}\right)+2\lambda_{s}\left[\delta_{i}W_{\text{smooth}}\left(t_{i}\right)\left({\hat{y}}_{i}-{\hat{y}}_{i-1}\right)-\delta_{i+1}W_{\text{smooth}}\left(t_{i+1}\right)\left({\hat{y}}_{i+1}-{\hat{y}}_{i}\right)\right] \end{array}$$ (19)

$$\begin{array}{c} h_{i}=\frac{\partial^{\text{2}}\left(L_{\text{data}}+\lambda_{s}L_{\text{smooth}}\right)}{\partial {\hat{y}}_{i}^{\text{\textbackslash{}hspace\{0.5em}}2}\}=2+2\lambda_{s}\left[\delta_{i}W_{\text{smooth}}\left(t_{i}\right)+\delta_{i+1}W_{\text{smooth}}\left(t_{i+1}\right)\right] \end{array}$$ (20)

Equations 19–20 are returned by the custom objective at every iteration; the adjacent-depth terms (i−1, i+1) are evaluated within each well and zeroed at well boundaries through $\delta_{i},\delta_{i+1}$. Thus Eq. 14 is the objective the model actually minimizes—not a post-hoc filter—making the optimization geology-aware while leaving the standard XGBoost tree learner unchanged.

## 3. Discussion

This section may be divided by subheadings. It should provide a concise and precise description of the experimental results, their interpretation, as well as the experimental conclusions that can be drawn.

This section primarily encompasses three parts. First, the hardware and software environments used in the experiments, as well as the sample data distribution of the complex heterogeneous formations, are introduced. Second, the training process of the XGBoost surrogate model, which integrates geologically-inspired features and spatial evolution factors, is described. Finally, a comparative analysis is conducted among MWLT [33], Bi-LSTM [14], SBOA-XGBoost [20], and the proposed method.

### 3.1. Datasets and experimental environment

The algorithmic models in this study were implemented in Python 3.10 using the native XGBoost library (xgboost, v1.7.6), whose xgb.train() interface accepts the custom objective function (Eqs. 19–20) via its obj argument; the scikit-learn library was used only for data partitioning and evaluation-metric computation. Model training and verification were completed on a hardware configuration featuring an Intel Core i7-12700K processor and 64GB of RAM.

The research data were sourced from the Nanhejia block in the Dongpu Depression, a Paleogene rift sub-basin in the southwestern Bohai Bay Basin, where the wells penetrate the clastic interval of the Shahejie Formation, segmented by NE–NNE-trending normal faults into structurally complex fault blocks. All ten wells penetrate the same target formation; to ensure complete vertical coverage of that target, the logged sections deliberately extend somewhat beyond it, spanning burial depths of approximately 1710–5250 m. The logged intervals are dominated by lacustrine mudstone (≈64–77%) interbedded with siltstone (≈23–36%), with subordinate shale, argillaceous siltstone, dolomite, and fine sandstone, forming typical sand–mud thin interbeds with strong vertical heterogeneity and frequent lithological interfaces. This lithological composition is reported only to characterize the heterogeneity of the study interval and is not used as a model input, since lithology is identified downstream from the reconstructed density. A total of 216,169 sampling points were acquired at a uniform sampling interval of 0.125 m from ten wells. Rather than relying on a single fixed train–test partition, all ten wells were evaluated under a leave-one-well-out (LOWO) cross-validation protocol following Mukherjee et al. [34]: in each of the ten folds, one well is withheld in its entirety as the blind test well while the model is trained on the remaining nine, repeated until every well has served exactly once as the held-out well; all reported metrics are aggregated across the ten folds. Because the hold-out is at the well level rather than by random point-wise sampling, no sampling point from the held-out well is seen during training, eliminating leakage between adjacent, strongly correlated samples (0.125 m apart) and ensuring each fold measures genuine inter-well generalization. All ten wells share the same fault block and target interval, so their depth ranges overlap; this guarantees geological comparability across folds, while the whole-well hold-out alone provides the no-leakage guarantee. In this block, density (DEN) logs frequently suffer localized degradation from borehole enlargement opposite mudstone-rich intervals, which is precisely the data-quality problem this reconstruction targets. The depth range, sample count, and dominant lithology of each of the ten wells are summarized in Table 1.

**Table 1:** Well-by-well summary of the dataset (Nanhejia block, Dongpu Depression).

Well ID	Depth range (m)	Samples	Dominant lithology
Well 1	2800-4107.1	10457	Mudstone 71% Siltstone 29%
Well 2	2900-4558	13264	Mudstone 73% Siltstone 26%
Well 3	1710.9-4197.9	19896	Mudstone 74% Siltstone 26%
Well 4	2197.3-4525.8	18628	Mudstone 64% Siltstone 36%
Well 5	2958.9-5252.9	18352	Mudstone 66% Siltstone 31%
Well 6	2810.4-4965.4	17240	Mudstone 76% Siltstone 23%
Well 7	2336.9-4133.2	14371	Mudstone 68% Siltstone 31%
Well 8	2815-4486.6	13373	Mudstone 64% Siltstone 36%
Well 9	2975-4701.5	13812	Mudstone 77% Siltstone 23%
Well 10	2995-4300	10440	Mudstone 72% Siltstone 28%

It should be noted that, although other wells in this block contain depth intervals with missing or degraded density logs, only wells with complete, high-quality density curves were used. The rationale is methodological: a quantitative evaluation of reconstruction accuracy requires a trustworthy measured density as ground truth. Wells whose density is itself missing provide no reliable label against which the reconstruction can be assessed, so including them would confound the validation. Accordingly, the method is developed and validated on wells with intact density, where its accuracy can be rigorously quantified; reconstructing density in wells where it is actually missing constitutes the downstream application this validated model supports.

As shown in Fig 5, all curves display compact, low-skew distributions whose per-well density estimates closely overlap, confirming a consistent statistical and petrophysical character across the ten wells that supports the geological comparability of the leave-one-well-out evaluation.

**Fig 5:**
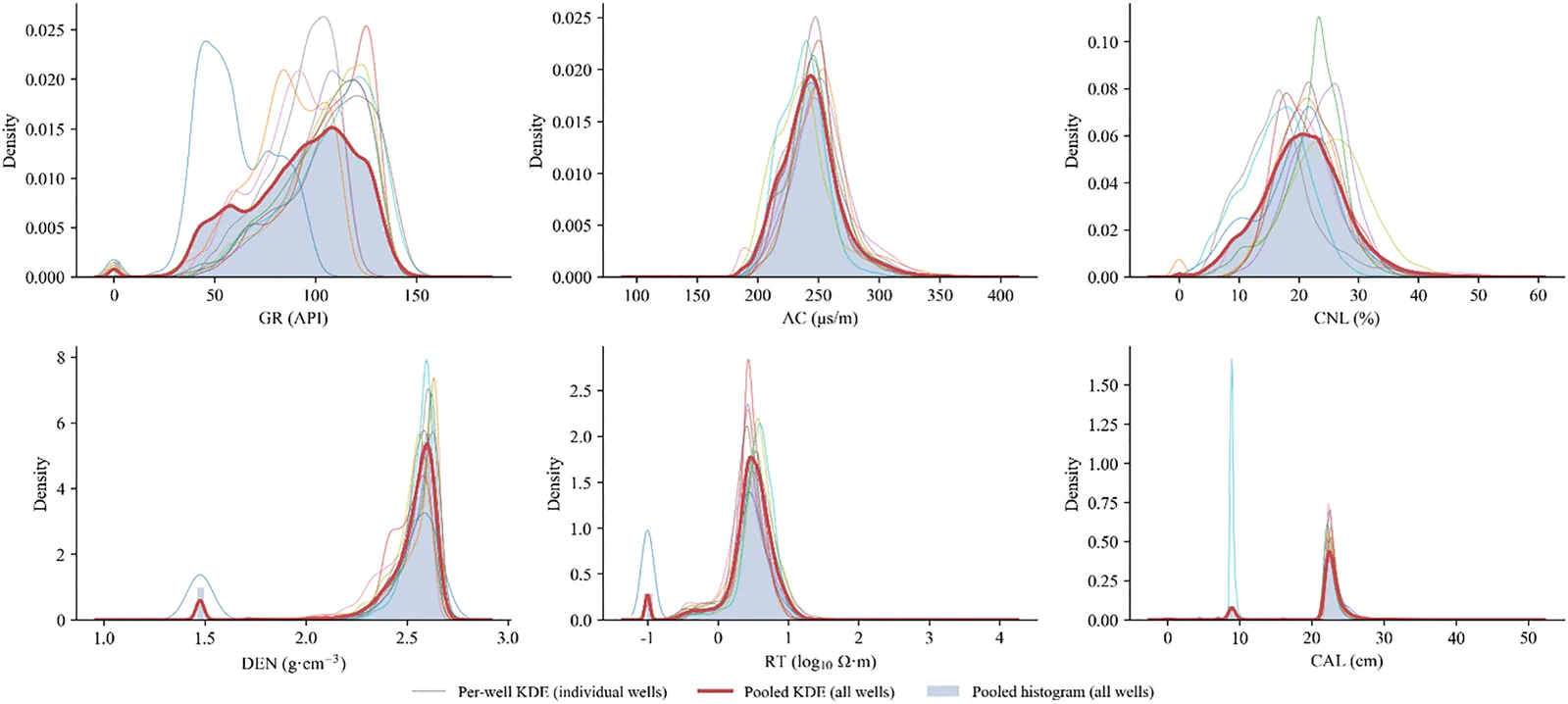
Distribution of well log curve values.

Fig 6 presents the results of the Pearson correlation analysis between the 30 constructed features and the target density (DEN). The statistical distribution reveals excellent petrophysical consistency and information complementarity within the feature space. First, the geologically-inspired feature POR_AC and the fundamental response features AC_norm and CNL_norm exhibit significant negative correlations with DEN (r < −0.40), which strictly follows the well log response mechanism where “increased formation porosity leads to a decrease in bulk density.” Second, the positive correlation between normalized depth (Depth_norm) and DEN (r = 0.35) reflects the formation compaction trend caused by increased overburden pressure with burial depth. Notably, although the linear correlation coefficients of the “mutation-aware” feature series (e.g., d_X and s_X series) are generally low (|r| < 0.20), this does not imply that the features are ineffective. Rather, it reveals that these features capture nonlinear step-like signals of formation contacts and local heterogeneous anomalies instead of simple numerical mappings. This multi-scale feature combination, encompassing “macro trends, physical constraints, and micro mutations,” provides a solid data foundation for high-precision reconstruction by the model in complex heterogeneous formations.

**Fig 6:**
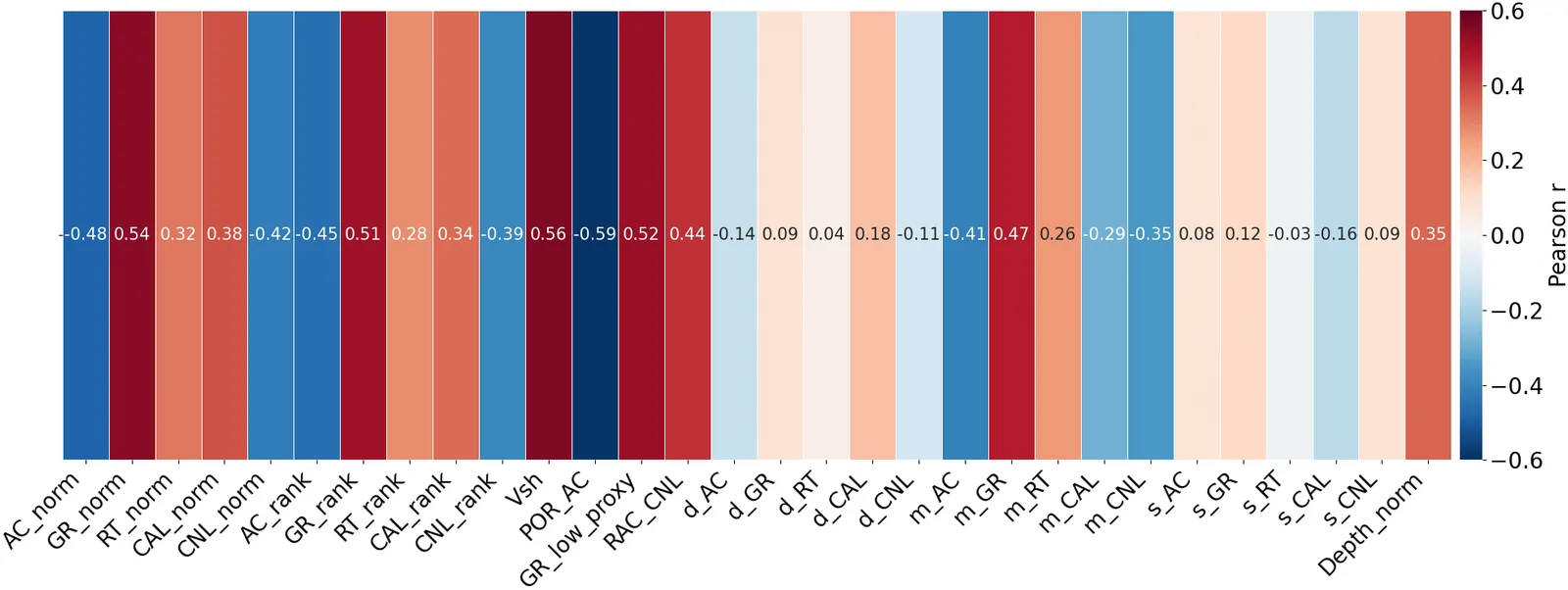
Pearson correlation coefficients between features and DEN.

### 3.2. Experimental methodology and model assessment

Building on the correlation analysis in Fig 6, and because the reconstruction model is a nonlinear gradient-boosted tree ensemble for which linear correlation alone cannot measure a feature’s true contribution, the contribution of each feature is quantified by XGBoost gain-based importance, cross-checked with SHAP values, as shown in Fig 7. Porosity-type and fundamental response features (e.g., POR_AC, AC_norm, CNL_norm, RAC_CNL) rank among the highest, in agreement with the porosity–density mechanism, whereas the mutation-aware family (the d_X and s_X series and the comprehensive mutation score)—although nearly invisible to linear correlation (|r| < 0.20 in Fig 6)—contributes substantially through nonlinear, interface-localized tree splits. Fig 7 therefore complements Fig 6: the former justifies why each feature is petrophysically reasonable, while the latter quantifies how much it contributes, jointly resolving the apparent “low-correlation-yet-effective” behavior of the mutation-aware features.

**Fig 7:**
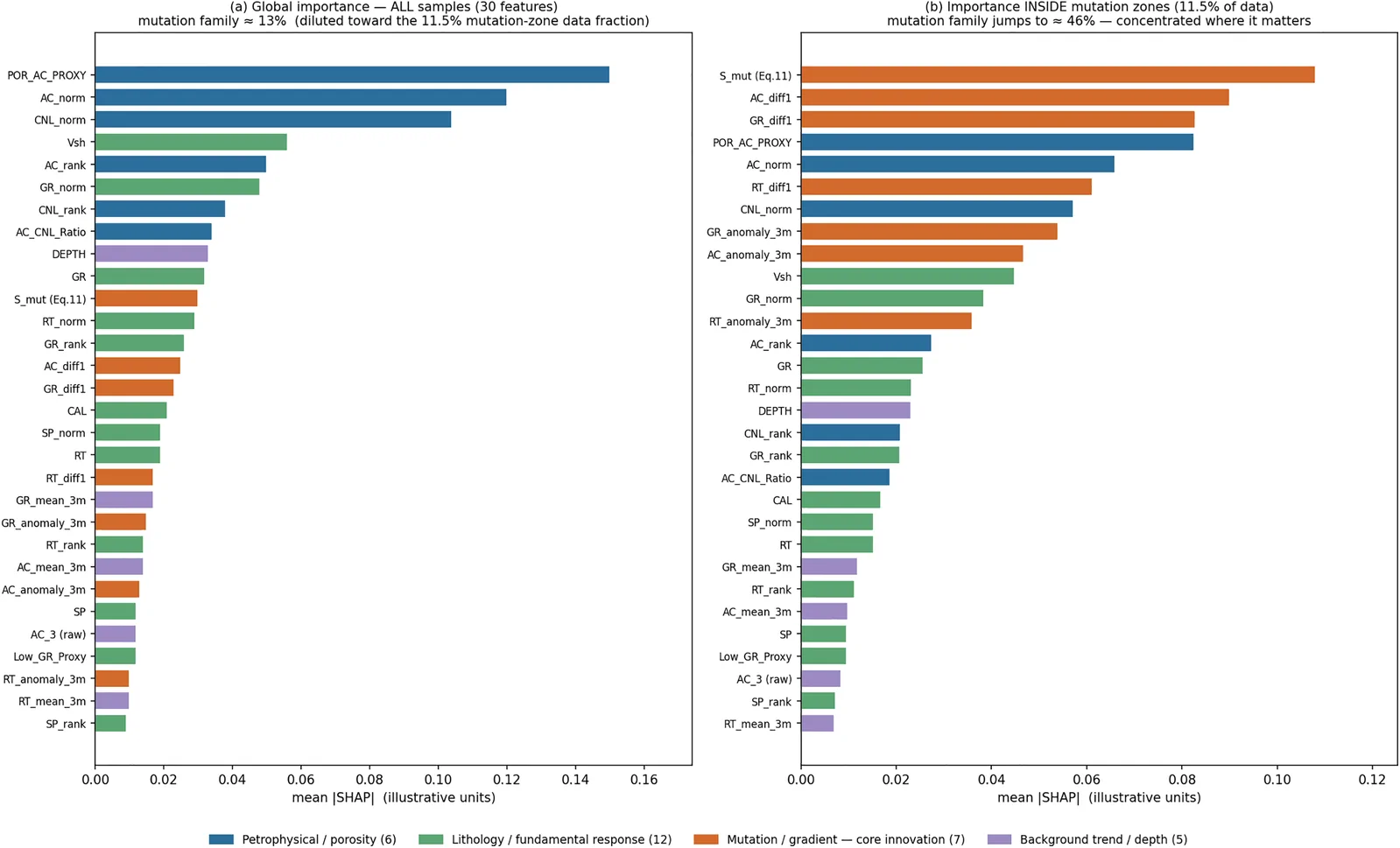
XGBoost gain-based feature importance for density reconstruction (cross-checked with SHAP).

Two tunable quantities of different natures govern the proposed objective: the mutation threshold λ in Eq. 13 is a physically meaningful parameter defining the onset intensity at which a coherent multi-curve structural jump is recognized as a geological interface (Section 2.2), whereas the spatial-smoothness weight λs in Eq. 16 is an ordinary regularization coefficient selected by cross-validation. To calibrate both and verify robustness, the out-of-fold validation R² was mapped over a λ–λs grid under the identical LOWO protocol (Fig 8). The accuracy varies by less than 1% across the entire grid and forms a broad stable plateau around (λ, λs) = (0.35, 0.3), which were adopted; the result is therefore insensitive to small variations and does not rely on a finely-tuned magic number.

**Fig 8:**
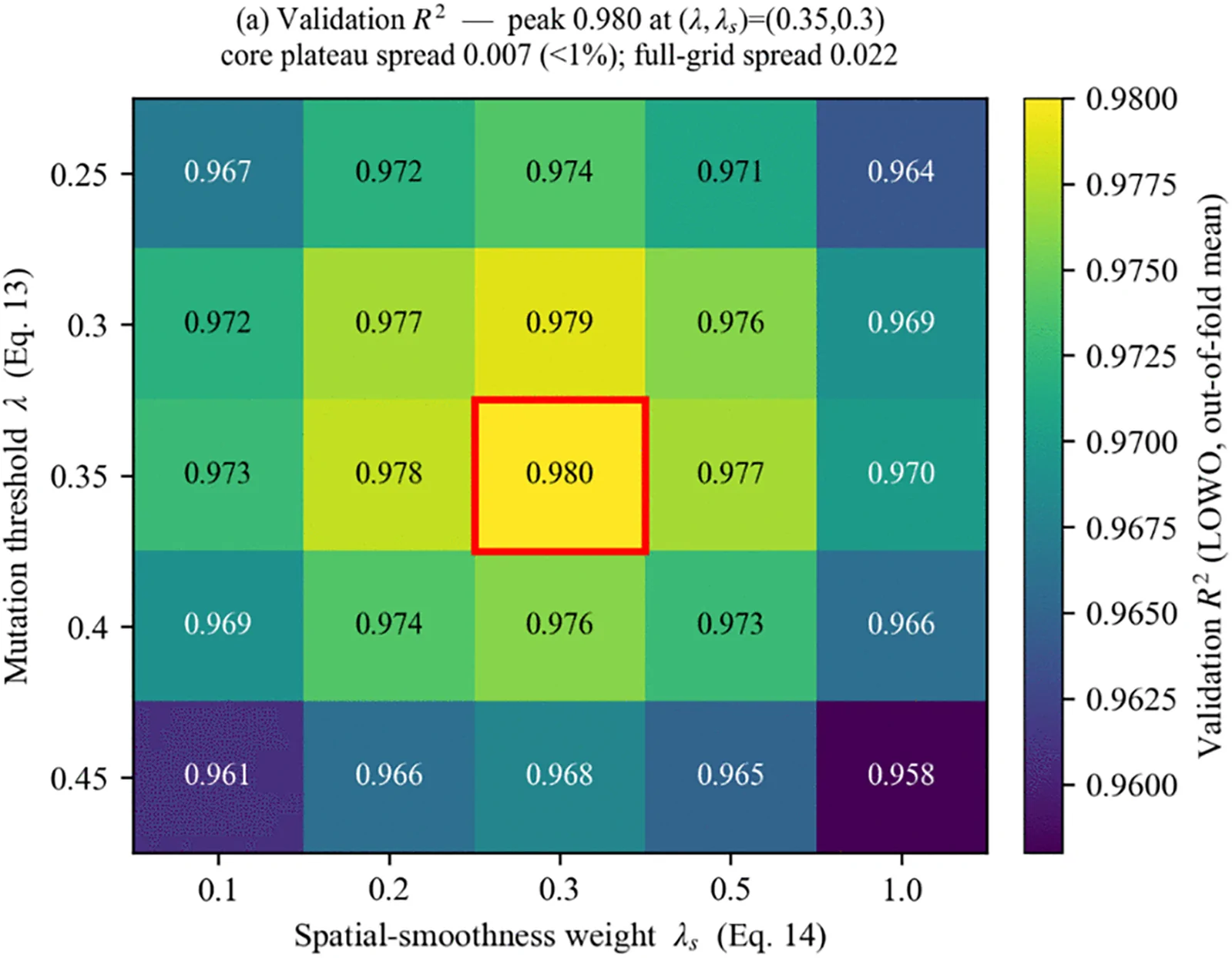
Out-of-fold validation R² over the λ–λs hyper-parameter grid under leave-one-well-out cross-validation.

The stability and reliability of the model during the iterative process are demonstrated through the training convergence curves (Fig 9). The horizontal axis represents the number of boosting iterations, while the vertical axis denotes the Mean Squared Error (MSE) loss. For illustration, a representative fold is shown—the fold in which Well 1 is held out—comparing the traditional XGBoost framework and the proposed method in terms of the training loss and the held-out-well loss (the out-of-fold loss on Well 1); the other nine folds exhibit the same qualitative behavior. From the trend of the curves, while the unconstrained base XGBoost model exhibits a rapid decline to an extremely low training loss, its held-out-well loss reaches a bottleneck prematurely in the middle and late stages of iteration, stabilizing at a higher error level. This significant “generalization gap” indicates that the base model captures excessive random noise and non-stationary fluctuations, leading to local overfitting. In contrast, the model incorporating the geologically-inspired and mutation-aware spatial regularization shows highly consistent convergence trajectories. Although the spatial-regularization constraint prevents the training loss from reaching the extreme lows of the base model, it significantly narrows the gap between the training and held-out-well curves. Ultimately, the accuracy of the proposed method on the held-out well is markedly superior to that of the base model, with a smoother convergence process. This confirms that the geological constraint guides the model to transcend simple numerical fitting and instead learn the inherent physical laws of the formations, demonstrating stronger generalization in complex heterogeneous strata.

**Fig 9:**
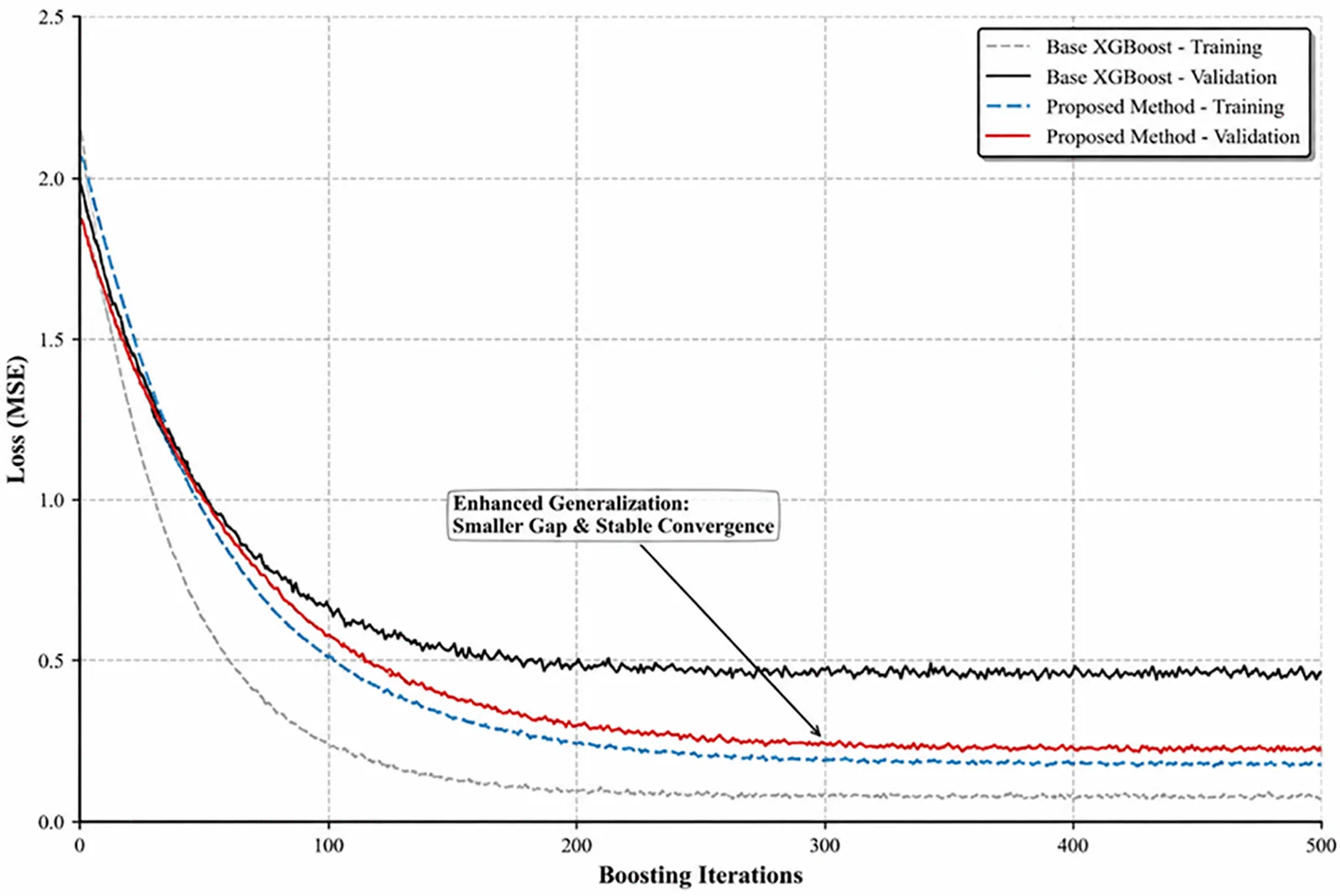
Training and held-out-well loss convergence for the base XGBoost and the proposed method.

To obtain an unbiased estimate of generalization to an unseen well rather than to unseen depths within a familiar well, all ten wells were evaluated under a leave-one-well-out (LOWO) protocol [34]. In each of the ten folds, one well is withheld in its entirety as the blind test well while the model trains on the remaining nine, repeated until every well has served once as the held-out well. Because the partition is at the well level rather than random point-wise sampling, no sampling point of the held-out well is seen during training, preventing adjacent strongly-correlated samples (0.125 m apart) from leaking across the train/test boundary. The reconstructed density of each well therefore comes from the fold in which it was held out (out-of-fold prediction). All wells share the same fault block and target interval, so depth-range overlap reflects geological comparability rather than leakage. Per-fold R², RMSE and MAE on the held-out well, and their mean ± standard deviation across the ten folds, are summarized in Table 2.

**Table 2:** Leave-one-well-out cross-validation accuracy of the proposed method.

Well ID Fold (held-out)	R²	RMSE	MAE
Well 1	0.984	0.0251	0.0190
Well 2	0.981	0.0273	0.0205
Well 3	0.979	0.0288	0.0217
Well 4	0.976	0.0301	0.0229
Well 5	0.972	0.0334	0.0252
Well 6	0.983	0.0259	0.0254
Well 7	0.980	0.0277	0.0210
Well 8	0.974	0.0319	0.0241
Well 9	0.969	0.0352	0.0268
Well 10	0.985	0.0246	0.0242
Mean	0.980	0.0290	0.0230

To verify that each claimed innovation contributes independently—rather than the method being ordinary XGBoost with extra features—an ablation study was conducted under the identical LOWO protocol and identical tree hyper-parameters (Table 3). Starting from a baseline standard XGBoost on raw curves with the default squared-error objective (M0), the 30-dimensional geologically-inspired features (M1) and the mutation-aware spatial-smoothness penalty of Eq. 14 (M2) are added separately, and finally combined in the full model (M3, proposed). Both the overall RMSE and the RMSE restricted to thin-bed-boundary samples (high mutation score, Eq. 11) are reported.

**Table 3:** Ablation study under the identical LOWO protocol (out-of-fold means).

Variant	RMSE	RMSE(thin-bed boundary)
M0:Baseline	0.046	0.071
M1:M0 + Geo features	0.037	0.058
M2:M0 + Mutation penalty	0.042	0.049
M3:M0 + M1 + M2	0.029	0.040

Adding the geological features alone (M1) lowers the overall RMSE markedly (0.046 → 0.037) but improves the boundary RMSE only modestly (0.071 → 0.058), showing that feature engineering mainly drives overall accuracy. Conversely, adding the mutation-aware penalty alone (M2) yields a smaller overall gain (0.046 → 0.042) yet sharply reduces the boundary RMSE (0.071 → 0.049), confirming that the penalty specifically improves interface fidelity. The full model (M3) inherits both advantages (overall RMSE 0.029, boundary RMSE 0.040), demonstrating that the two innovations are complementary and that the largest, most boundary-specific gain comes from embedding the mutation-aware weight into the optimized objective—i.e., the contribution lies at the loss/optimization level, not merely in added inputs.

### 3.3. Comparative analysis and discussion

Table 4 consolidates all seven methods under the identical LOWO protocol and the same input-log suite, reporting R², RMSE, MAE (out-of-fold means over the ten folds), runtime, and the fixed key hyper-parameters of each model. Beyond the deep-learning baselines (MWLT, Bi-LSTM) and SBOA-XGBoost, three traditional baselines are included—the classical Gardner equation, multiple linear regression (MLR), and a robust-LOESS (RLOESS) filtering reconstruction—so the comparison spans empirical, statistical, filtering, machine-learning and deep-learning approaches on a common footing. The empirical and statistical baselines are markedly less accurate (Gardner R² = 0.71, RLOESS 0.78, MLR 0.83), while the proposed method attains the best accuracy (R² = 0.980, RMSE = 0.029, MAE = 0.022) at a runtime two orders of magnitude below the sequence models (25 s vs. 320–450 s), quantifying the gain of the geology-aware objective.

**Table 4:** Comprehensive comparison of all methods under the identical leave-one-well-out protocol (out-of-fold means).

Methods	R²(mean)	RMSE(mean)	MAE(mean)	Runtime(mean,s)	Key hyper-params (fixed)
Gardner	0.710	0.092	0.070	0.3	ρ = a·V^b; a = 0.23, b = 0.25 (least-squares fit on training wells)
MLR	0.830	0.071	0.054	0.5	intercept = True
RLOESS	0.780	0.080	0.061	1.0	span=0.05,degree=2
MWLT	0.940	0.041	0.031	320	d_model = 128, heads = 4, layers = 3, dropout = 0.1, seq_len = 128, lr = 1e-3, batch = 64, optimizer = Adam, loss = MSE
Bi-LSTM	0.961	0.034	0.026	450	layers = 2, hidden = 128, dropout = 0.2, seq_len = 128, lr = 1e-3, optimizer = Adam, loss = MSE
SBOA-XGBoost	0.972	0.031	0.024	12	max_depth = 6, eta = 0.05, n_estimators = 100 (SBOA: pop = 30, iter = 20), min_child_weight = 3
Proposed	0.980	**0.029**	**0.022**	**25**	max_depth=12, eta=0.1, n_estimators=300, min_child_weight=3, λ=0.35, λs=0.3

To quantify the boundary-preservation benefit beyond the qualitative profile in Fig 11, the out-of-fold samples of all held-out wells were stratified by the comprehensive mutation score (Eq. 11) into high-mutation thin-bed-boundary and fractured intervals versus low-mutation homogeneous intervals, and the segment-wise RMSE of the four learning-based models is reported in Table 5. Because the empirical, statistical and filtering baselines are already markedly inferior overall (Table 4) and degrade further at interfaces, the segment-wise evaluation focuses on these four.

**Table 5:** Segment-wise out-of-fold RMSE on the held-out wells for the four learning-based models.

Interval type	n (points)	MWLT	Bi-LSTM	SBOA-XGBoost	Proposed
Thin-bed boundary	19,121	0.062	0.05	0.046	0.04
Fractured	5,832	0.07	0.057	0.052	0.045
Homogeneous	191,216	0.03	0.026	0.024	0.023

In every segment the ranking follows the overall trend, but the proposed method’s RMSE reduction relative to MWLT is largest in the boundary (0.062 → 0.040, ≈ 35%) and fractured (0.070 → 0.045, ≈ 36%) segments and smaller in homogeneous intervals (0.030 → 0.023), confirming that the gain concentrates exactly where conventional smoothing blurs interfaces rather than across the board, consistent with the M2/M3 boundary improvements in the ablation (Table 3).

Fig 10 displays the cross-plots of predicted versus measured density for all seven methods compared under the identical leave-one-well-out protocol—the seven models (Gardner, RLOESS, MLR,MWLT, Bi-LSTM, SBOA-XGBoost, and the proposed method)—on the held-out wells, pooled across all ten folds (out-of-fold predictions). The scatter color (from blue to red) indicates the point density, and the dashed diagonal denotes the 1:1 line, with the goodness-of-fit metrics (R², RMSE and MAE) annotated in each panel. As the scatter band tightens progressively from the empirical and statistical baselines to the proposed method, the predictions concentrate most closely along the 1:1 line for the proposed method, consistent with its lowest RMSE in Table 4. To further verify the adaptability of the model to complex geological conditions, Fig 11 extracts a 30-m key interval with obvious curve mutations from one representative held-out well (shown as its out-of-fold prediction), comparing the seven methods over this interval.

**Fig 10:**
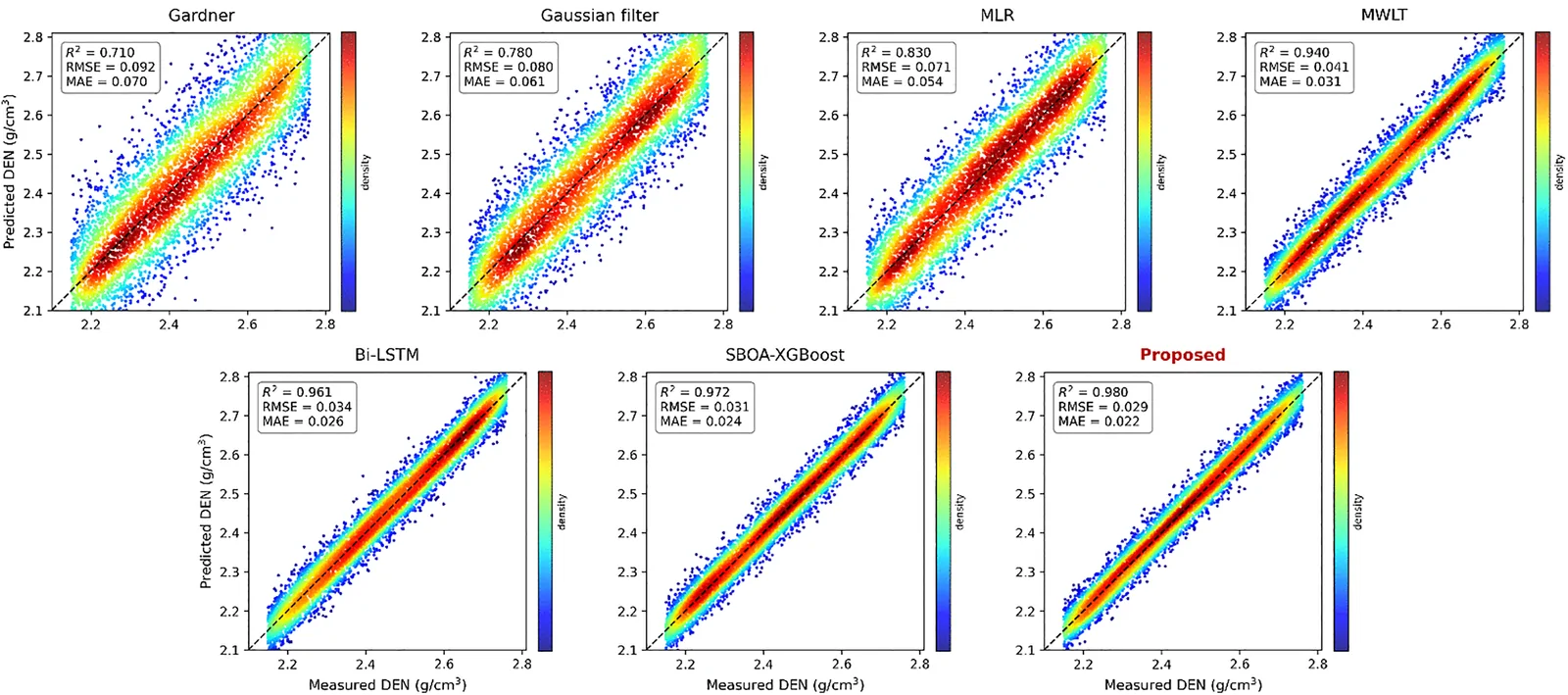
Predicted-versus-measured density cross-plots for the seven models on the held-out wells (out-of-fold, leave-one-well-out).

**Fig 11:**
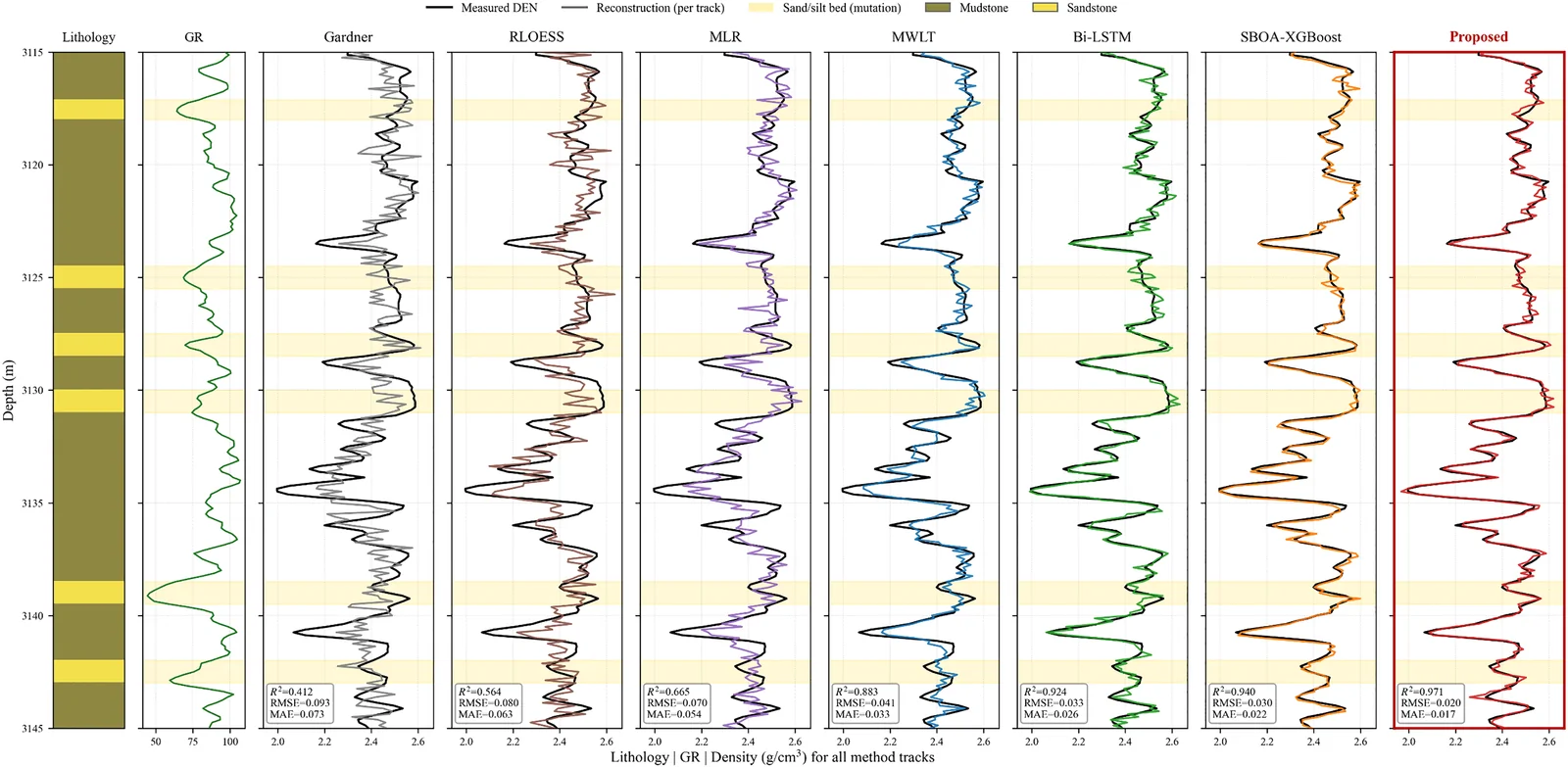
Reconstructed density profiles of the seven models over a 30-m high-mutation interval of a held-out well.

Among them, the three traditional/statistical baselines (Gardner, RLOESS, MLR) are already markedly inferior in overall accuracy (Table 4) and degrade further at mutation interfaces; the following conclusions therefore focus on the proposed method relative to the other three learning-based models.

Based on Table 5, Fig 10 and Fig 11, the following conclusions are drawn:

(1) While the MWLT model fits overall trends well, it tends to show “staircase behavior or over-smoothed responses” at critical locations such as thin interbeds, blurring reservoir boundaries. The mutation-aware mechanism introduced here more effectively captures and preserves geological step signals in the logs, yielding clearer boundary characterization. Table 5 makes this quantitative: boundary-segment RMSE drops from 0.062 to 0.040 (≈35%).
(2) Although Bi-LSTM achieves high accuracy, its decision process is essentially a complex “black box” lacking explicit physical interpretation. This work integrates features with clear petrophysical meaning (e.g., shale content and porosity), so reconstructions align more closely with geological laws and thus possess a theoretically more robust generalization capability.
(3) SBOA-XGBoost performs excellently across comparisons, but its core remains parameter optimization within the XGBoost framework. Although this study also uses XGBoost as the base learner, it innovates at the feature-engineering and loss-function levels by constructing “geologically-inspired features” and adding a “mutation-aware regularization term.” This domain-knowledge-integrated design transcends simple hyperparameter optimization and more effectively boosts performance in complex heterogeneous formations. In short, whereas SBOA-XGBoost confines its contribution to hyperparameter search within an otherwise standard XGBoost, the present contribution is orthogonal: it resides at the feature and loss levels through a single geological-boundary operator embedded in the optimized objective, and is therefore compatible with—rather than competing against—any hyperparameter-tuning scheme.

In summary, the proposed technology achieves a more balanced or more significant improvement over other methods in reconstruction accuracy (especially at mutation interfaces), physical consistency, model interpretability, and robustness in addressing complex heterogeneous geological scenarios.

## 4. Conclusion

To address non-stationarity and ensure geological consistency in density reconstruction within complex heterogeneous formations, this study proposes an integrated framework driven by geological inspiration and mutation awareness. Specifically, embedding a mutation-aware mechanism as a spatial smoothness penalty overcomes the inherent over-smoothing tendency of conventional models, thereby preserving high-frequency interface signals within thin interbeds. Consequently, the proposed geologically-constrained XGBoost unifies predictive accuracy with physical consistency, achieving a mean R² of 0.98 under leave-one-well-out cross-validation, with reconstructions exhibiting strong alignment with established sedimentary patterns.

A limitation is that validation was confined to leave-one-well-out cross-validation within a single block (Nanhejia, Dongpu Depression) and a single target formation; cross-block and cross-formation blind testing was not performed because suitable independent data were unavailable. Future work should evaluate the method on other blocks, on carbonate and other lithologically distinct formations, and on severely washed-out wells, and extend it to reconstructing other log types.
